# P-163. Trimethoprim-Sulfamethoxazole is Associated with Decreased Mortality Compared to Fluoroquinolones for Secondary Prophylaxis of Spontaneous Bacterial Peritonitis: A Global Network Analysis

**DOI:** 10.1093/ofid/ofaf695.387

**Published:** 2026-01-11

**Authors:** Urvya Iyer, Mikaela Nikkola Jara-Tantoco, Alejandro Delgado

**Affiliations:** Einstein Medical Center Philadelphia, Philadelphi, Pennsylvania; Jefferson-Einstein Hospital, Philadelphia, Pennsylvania; Einstein Medical Center Philadelphia, Philadelphi, Pennsylvania

## Abstract

**Background:**

Historically, fluroquinolones and trimethoprim-sulfamethoxazole (TMP-SMX) have been used for secondary prophylaxis for spontaneous bacterial peritonitis (SBP). Previous studies with smaller populations have shown that there is no difference in mortality and efficacy between the two.

We aimed to look at mortality rates and other adverse events related to taking fluoroquinolones versus TMP-SMX for SBP prophylaxis in a global retrospective cohort study.

**Figure:**
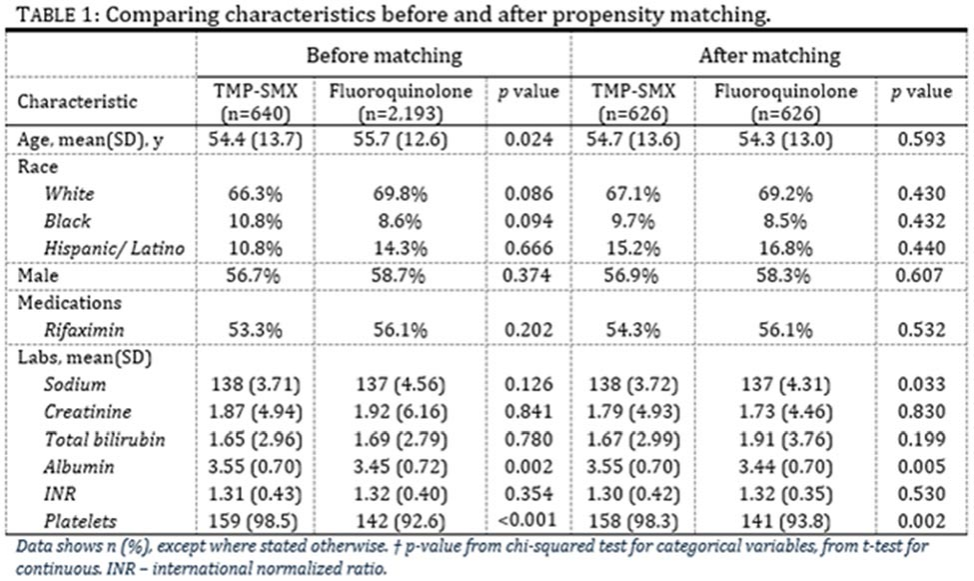

**Figure:**
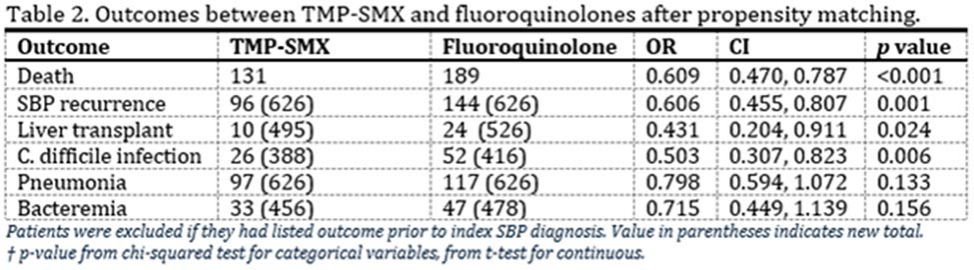

**Methods:**

Patients were selected in the TrinetX database from January 1, 2015 to April 1, 2025. Inclusion criteria included age over 18 and index diagnosis of SBP. Patients with death or liver transplant within one month were excluded. Those with prescription of either ciprofloxacin or norfloxacin within four months were then added and excluded from the TMP/SMX group, and vice versa. Patients without any documented labs between 18 and 24 months after diagnosis were excluded.

The two groups were then propensity matched for values listed in Table 1. Odds ratio and Kaplan-Meier survival analysis was then conducted on rates of death, recurrent SBP, C. difficile, bacteremia, pneumonia, and liver transplant within two years following index diagnosis.

**Figure 1.**
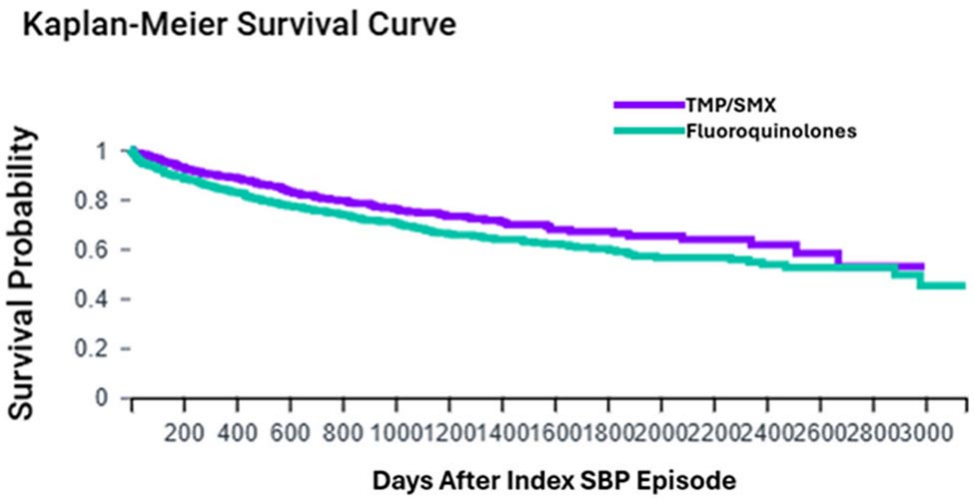
Kaplan-Meier survival curve comparing mortality in patients who received TMP-SMX versus those who took fluoroquinolones for SBP prophylaxis.

**Results:**

Prior to propensity matching, baseline demographics were similar (Table 1). After matching, we had 626 patients in each cohort. We found decreased mortality in the TMP/SMX group (Table 2) with 21% compared to 30% in the fluoroquinolone group. Kaplan Meier analysis is displayed in Figure 1. Secondary outcomes showed decreased rates of recurrent SBP, C. diff, bacteremia, and pneumonia in the TMP/SMX group as well, although only SBP and C. diff are significant. It also showed a higher rate of liver transplant in the fluoroquinolone group.

**Conclusion:**

Ultimately, our analysis found decreased mortality and infection rates in our cohort prescribed TMP/SMX compared to fluoroquinolones.

Our advantages lie in the width and breadth of our study population. Our primary limitation is that our dataset is based on ICD-10 codes. We mitigated follow-up bias by ensuring follow-up in the chart for at least 18 months; however, diagnoses other than death remain dubious without further clinical information. More detailed RCTs or retrospective studies must be performed.

**Disclosures:**

All Authors: No reported disclosures

